# Genome Wide Prediction, Mapping and Development of Genomic Resources of Mastitis Associated Genes in Water Buffalo

**DOI:** 10.3389/fvets.2021.593871

**Published:** 2021-06-18

**Authors:** Sarika Jaiswal, Jaisri Jagannadham, Juli Kumari, Mir Asif Iquebal, Anoop Kishor Singh Gurjar, Varij Nayan, Ulavappa B. Angadi, Sunil Kumar, Rakesh Kumar, Tirtha Kumar Datta, Anil Rai, Dinesh Kumar

**Affiliations:** ^1^Centre for Agricultural Bioinformatics, Indian Council of Agricultural Research (ICAR)-Indian Agricultural Statistics Research Institute, New Delhi, India; ^2^Indian Council of Agricultural Research (ICAR)-Central Institute for Research on Buffaloes, Hisar, India; ^3^Animal Biotechnology Centre, Indian Council of Agricultural Research (ICAR)-National Dairy research Institute, Karnal, India

**Keywords:** GWAS, mammary gland, markers, mastitis, TGP, water buffalo

## Abstract

Water buffalo (*Bubalus bubalis*) are an important animal resource that contributes milk, meat, leather, dairy products, and power for plowing and transport. However, mastitis, a bacterial disease affecting milk production and reproduction efficiency, is most prevalent in populations having intensive selection for higher milk yield, especially where the inbreeding level is also high. Climate change and poor hygiene management practices further complicate the issue. The management of this disease faces major challenges, like antibiotic resistance, maximum residue level, horizontal gene transfer, and limited success in resistance breeding. Bovine mastitis genome wide association studies have had limited success due to breed differences, sample sizes, and minor allele frequency, lowering the power to detect the diseases associated with SNPs. In this work, we focused on the application of targeted gene panels (TGPs) in screening for candidate gene association analysis, and how this approach overcomes the limitation of genome wide association studies. This work will facilitate the targeted sequencing of buffalo genomic regions with high depth coverage required to mine the extremely rare variants potentially associated with buffalo mastitis. Although the whole genome assembly of water buffalo is available, neither mastitis genes are predicted nor TGP in the form of web-genomic resources are available for future variant mining and association studies. Out of the 129 mastitis associated genes of cattle, 101 were completely mapped on the buffalo genome to make TGP. This further helped in identifying rare variants in water buffalo. Eighty-five genes were validated in the buffalo gene expression atlas, with the RNA-Seq data of 50 tissues. The functions of 97 genes were predicted, revealing 225 pathways. The mastitis proteins were used for protein-protein interaction network analysis to obtain additional cross-talking proteins. A total of 1,306 SNPs and 152 indels were identified from 101 genes. Water Buffalo-MSTdb was developed with *3-tier architecture* to retrieve mastitis associated genes having genomic coordinates with chromosomal details for TGP sequencing for mining of minor alleles for further association studies. Lastly, a web-genomic resource was made available to mine variants of targeted gene panels in buffalo for mastitis resistance breeding in an endeavor to ensure improved productivity and the reproductive efficiency of water buffalo.

## Introduction

Water buffalo (*Bubalus bubalis*) have been domesticated for more than 5,000 years, are also known as “Black Gold” due to their economic value. Its contribution to the Indian GDP in terms of milk, meat, horns, hides, leather, dairy products like cream, butter, yogurt, and cheese, power, plowing, and transporting people and crops have made buffalo an important animal resource in rural areas. India ranks first in world milk production due to the largest bovine population, where buffalo contributes 55%. The estimated world population of water buffalo is 208 million which is spread across over 77 countries in five continents with a major population (96%) in Asia (1). Water buffalo is an efficient converter of poor-quality forages into high quality milk and meat, making it a very valuable genetic resource for countries having “Low External-Input System” (2). Its efficiency of rumen fermentation (3) and nitrogen utilization (4) ability make it the animal of preference. However, intense selection for dairy productivity often results in increased levels of inbreeding, due to the limited number of bulls in the gene pool. Furthermore, climate change with a rising temperature humidity index (THI) has induced mastitis and many other diseases in major dairy animals including buffalo like retained placenta, metritis, ovarian cysts, claw diseases, milk fever, ketosis, and displaced abomasum (5–7).

Mastitis infections cause inflammation of the mammary gland and udder tissue, which is one of the most complex, costlier endemic diseases of dairy animals. It occurs as an immune response to invasion by a group of bacterial species in the teat canal. This may also occur due to chemical, mechanical, or thermal injury to the udder. This disease causes huge economic loss due to lesser milk production, premature culling, veterinary costs, and the risk of drug residues (8). Thus, there is a need to reduce mastitis to increase profitability and health (9).

Reduced milk production in buffalo mastitis costs US$8.80 per lactation (10). The direct loss happens due to a reduction in milk yield (70%), milk discard after treatment (9%), cost of veterinary services (7%), and premature culling (14%). There is also an indirect loss by adverse reproductive efficiency because of an imbalance in luteinizing hormone (LH) and estradiol that leads to failure of ovulation (11). In India, a total annual loss due to mastitis (clinical and subclinical) in buffalo has been estimated at US$526 million (10). Both clinical and subclinical mastitis is associated with mammary inflammation, a difference seen in clinical detectable and undetectable changes, respectively. With clinical mastitis, clinical visible signs are accompanied in the milk or mammary parenchyma. In subclinical mastitis conditions, a specific diagnostic test is performed (12).

Antibiotic treatment is effective in only 60% of cases of mastitis, which is due to an increase in b-lactamase producing organisms (10). The indiscriminate use of antibiotics has also led to a rise in resistant bacterial strains in Indian buffalo (13). All these are of global concern which is highly alarming due to the rise in AMR (antimicrobial resistance) and HGT (Horizontal Gene Transfer) of ARG (Antibiotic Resistance Genes) (14). The traditional breeding approach for the reduction of mastitis has not been effective due to its low heritability and its unfavorable genetic correlation with milk yield (15).

Recent studies on GWAS of mastitis disease in bovines have revealed that breed difference, sample size, and minor allele frequency, may lower the power to detect the disease-associated SNPs (16). Though universal NGS array based GWAS and genomic selection have been successful in animal breeding for better genetic gain of productivity traits, mastitis resistance breeding has still had limited success. GWAS has a major limitation in that it implicates the entire genome in disease predisposition where most association signals reflect variants/genes with no direct biological relevance to the disease (17). A control group of animals may contain unexposed individuals with susceptible genotypes. In such situations, a candidate gene approach tends to have greater statistical power than GWAS (18). GWAS can miss data on intron retention, which works in disease resistance, as reported in water buffalo (19). The success of the candidate gene approach depends on the correct choice of targeted gene panel (TGP) for SNP discovery, focusing on disease associated pathways in the investigation (20).

In genome assisted selection, the candidate gene approach using TGP and GWAS complement each other, rather than being contradictory. TGP can be used pragmatically in dissecting the genetics of complex disease by the mining of extremely low frequency alleles required in case-control association analysis. This ensures that minor novel alleles are not missed, which are not present in the prefabricated SNP array/chip (21, 22). TGP approach offers multifold advantages like sequencing at higher depth (500–1000X coverage) than the whole genome/exome sequencing, allowing the discovery of extremely rare variants which are potentially the most valuable alleles for association studies (23, 24). It is rapid and cost effective, especially in the case of association studies where the sample size is limited and mining of causative mutations in a single assay is imperative (25). Variant mining of TGP can be done either by direct amplicon sequencing (if TGP <50) or by target enrichment by magnetic beads followed by NGS data generation (26).

Post-GWAS, prioritization of candidate genes for mastitis resistance breeding in cattle has been reported (27). A computational approach has been reported to identify the candidate genes for disease association studies (28). A genomic resource database for cattle candidate genes and genetic markers for milk production and mastitis has also been developed (29). However, there is no targeted gene panel genomic resource of water buffalo for mastitis association studies. Recently available WGA with chromosome wise genomic data can be used for the development of genomic resources cataloging TGP. Since *taurine* and *babuline* genomes have extensively conserved milk and mastitis associated genes, comparative genomics approaches can quickly enrich buffalo genomic resource development.

The present work aims to develop a targeted gene panel of mastitis genes in water buffalo. It anticipates the prediction of mastitis associated candidate genes in the water buffalo genome using publically available bovine mastitis genes. It also aims to validate the RNA-Seq library in water buffalo, along with predicting biochemical pathways mediating this disease through the protein-protein network analysis. We present a web-genomic resource that is required for variant mining for future association studies of mastitis disease.

## Materials and Methods

### Mining and Mapping of Bovine Mastitis Associated Genes

An intensive literature search was carried out covering >40 literature across water buffalo (*Bubalus bubalis*) and cattle (*Bos taurus*) genes. Distinct terms associated with mastitis, *namely*, mastitis resistance, tolerance, and traits association were used as keywords for this search. These records were compiled and filtered to remove duplicates to finally fetch the genes associated with mastitis for further analyses in the present study.

Earlier studies on buffalo mastitis genes were reported even before the availability of the *de novo* genome assembly but then chromosomal number and position in the physical map were not known. By applying the NGS-based variant mining from TGP in a given buffalo population, the targeted genes or region of interest can be mapped. To accomplish this, the entire set of genes reported in *Bubalus bubalis* was downloaded from NCBI (30). Prior to further analyses, these were subjected to manual curation based on the organism specific term “*Bubalus bubalis*.” The finally filtered genes were used to map mastitis associated genes to obtain their genomic coordinates in the chromosome-wise genome assembly. In-house Perl scripts were used to map, based on gene symbol and its *alias*. Based on these parameters, mapping was accomplished for genes indicating features like gene symbol, gene ID, description, chromosome number, chromosome start and end location, strand orientation, and exon count.

### Validation of Predicted TGP Genes in the Water Buffalo Gene Expression Atlas

Since genes in TGP panels are computationally predicted using cattle genome, their expressional reliability was validated in the RNA-Seq data of water buffalo available in the gene expression atlas of 50 different tissues (31). This validation was done using an in-house generated Perl script.

### Functional Prediction of Mastitis Associated Candidate Genes

The function and pathway enrichment analysis of genes were performed using PANTHER (32) and DAVID v6.8 (Database for Annotation, Visualization, and Integrated Discovery) available at https://david.ncifcrf.gov/ (33, 34). It utilizes the Kyoto Encyclopedia of Genes and Genomes (KEGG) for pathway analysis and Gene Ontology (GO) analyses for functional prediction.

Annotation tool DAVID had *Bubalus bubalis* specific 91 genes. Since, in bovines, mastitis-associated genes are highly conserved, further analysis was done by taking advantage of this using cattle (*Bos taurus*) in PANTHER (http://pantherdb.org/data/). KEGG Mapper (Version 4.1), a mapping tool was used to reconstruct pathways (35). KEGG Orthology (KO) annotation was retrieved for key candidate genes and pathway reconstruction was performed specific to the *Bubalus bubalis* species.

### Prediction of Cross-Talking Proteins by Protein-Protein Network Analysis

The list of genome-based candidate genes was further enriched by computational prediction of mastitis disease associated proteins (DAP) using protein-protein interaction (PPI) analysis. The amino acid sequences of mastitis associated genes were retrieved from NCBI, using advanced search options with the keywords “mastitis” and organism “*Bubalus bubalis*” (36). The cross talk of candidate gene proteins with additional predicted putative candidate gene proteins were predicted computationally by PPI analysis using the STRING database (Version 11.0; https://string-db.org/) (37). This database scores and integrates the various sources of PPI after collecting these from the public domain and complements the information with computational predictions. STRING database aims at a comprehensive and objective global network, considering both, physical as well as functional interactions. Proteins are represented as nodes and the interactions among them are pictured as edges in the network. Furthermore, Cytoscape (Version 3.7.1) (38) was used to visualize the PPI network. To get the most inter-connected nodes from the constructed PPI network complex, additional Cytoscape plugins (MCODE and CentiScape) were used. To predict the extended interaction among proteins, the retrieved number of proteins was increased up to 100. All these interactions were predicted specific to *Bubalus bubalis* species.

### Evaluation of Mastitis TGP Based Variant Mining (SNP and Indel)

RNA-Seq data of mammary gland tissues (ERR315636) and Refseq of water buffalo (GCA_003121395.1) were downloaded from NCBI. The data was processed by FastQC (39) to check the quality. After the quality was checked low-quality reads and adapters were removed using the Trimmomatic tool V. 0.36 (40) by selecting the parameters of Phred quality score ≥20, Minimum reads length = 25 and Leading:4, Trailing:4 and Sliding-window:4:20. Filtered pair-end reads were aligned to the reference (genome assembly of water buffalo) using Burrows-Wheeler Aligner (bwa-0.7.17) tool (41, 42). The Sequence Alignment Map (SAM) file was converted to Binary Alignment Map (BAM) using SAMtools-1.9 (43) followed by sorting of BAM file. Variant calling analysis for analyzing genotype likelihoods was done with mpileup function with BCFtools 1.9 (44). SNPs were filtered at *p* < 0.05, read depth (d) ≥10, quality depth (Q) ≥30, MQ (minimum root mean square mapping quality) ≥40, and flanking sequence length 50. Genomic coordinates of SNPs and indels from the VCF file were mapped with coordinates of 101 genes using in-house generated shell scripting.

### Development of Web Resource: Water Buffalo-MSTdb (*WBMSTDb*)

**Figure 1:**
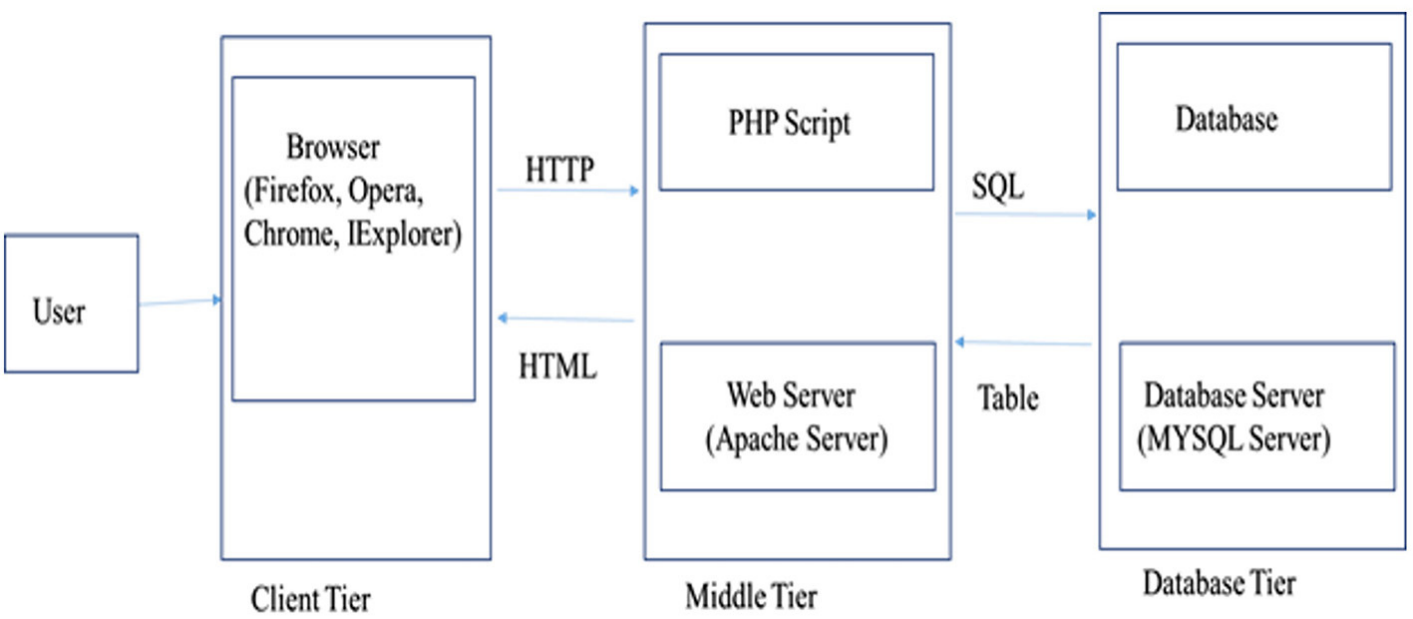
Three tier architecture representing client, middle and database tier of *WBMSTDb*.

Water Buffalo Mastitis Database (*WBMSTDb*) is an online relational database that catalogs information of bovine mastitis associated genes, their information along with the functions and annotations, predicted genes and their functions by close and other interaction PPI networks and *in-silico* SNPs and indels. The web resource also houses various pathways where these genes are known to be involved. It has been developed using “*three tier architecture*,” *namely*, a client tier, middle tier, and database tier (Figure 1). Client-end assists to display the information related to services available and build the front-end layer of the application for end-users of their customized search related to mastitis in buffalo. The database layer has been developed using PHP (Hypertext Preprocessor) which is an open-source server-side scripting language. The application layer, also known as the middle tier or logic tier takes the information from the presentation tier and controls the application's core functionality. Database tier has the information related to mastitis associated genes, gene pathways, proteins, and PPI, functions, and variants, etc. cataloged in MySQL database using SQL query language. *WBMSTDb* has been developed using LAMP (Linux-Apache-MySQL-PHP) technology and launched at Apache server.

## Results

### Mining and Mapping of Bovine Mastitis Associated Genes

A total of 159 mastitis associated genes were sourced from literature, taking water buffalo (*Bubalus bubalis*) and cattle (*Bos taurus*) as reference. After removing the duplicates and alternative gene alias, 129 genes associated with mastitis were used for further study. Out of 129 unique genes associated with mastitis, 120 genes were retrieved from highly diverse cattle breeds like Canadian Holsteins, Holstein, Holstein Friesian, and Brown Swiss, Chinese Holstein, Sanhe cattle, Chinese Simmental, Luxi Yellow, and Bohai Black. The remaining 9 genes were reported both in cattle breeds mentioned and in buffalo breeds like Murrah and Egyptian buffalo.

**Figure 2:**
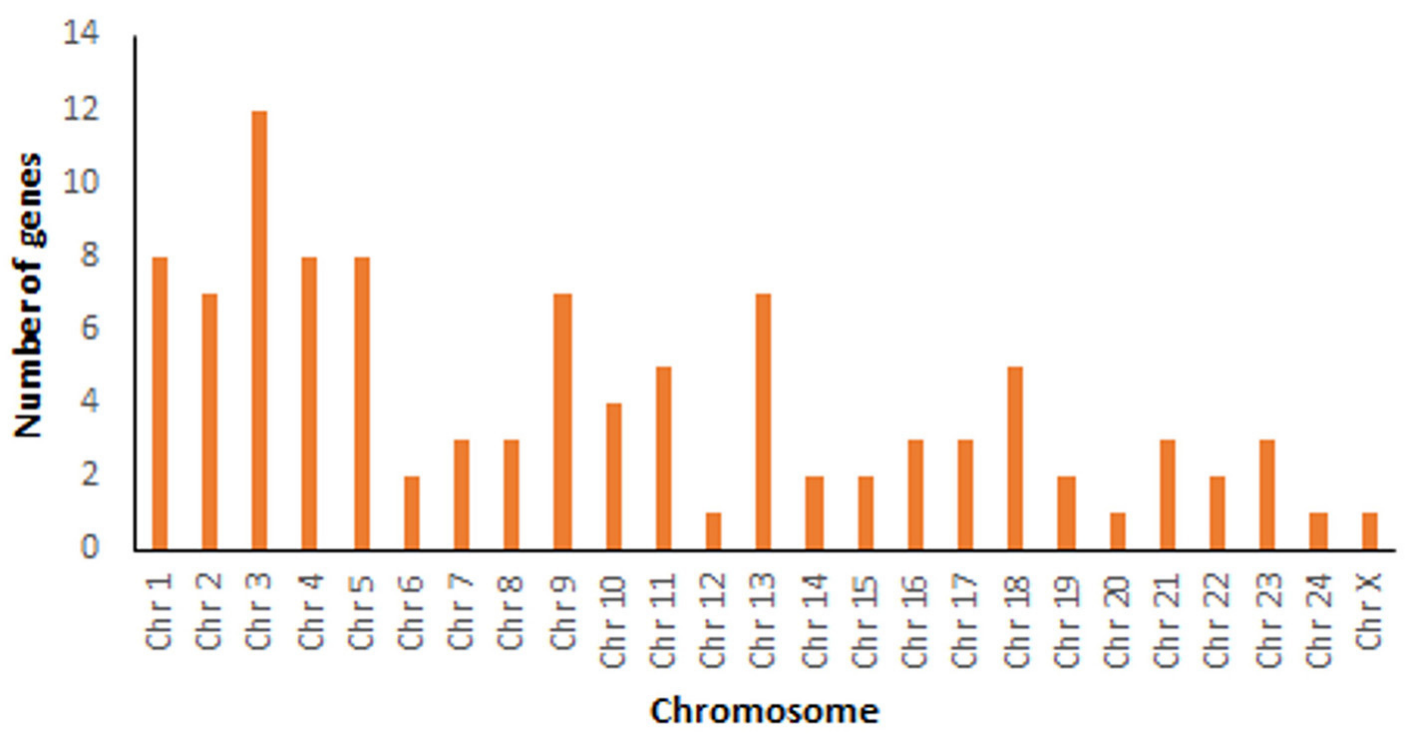
Chromosome-wise mapping of 101 mastitis associated genes in *Bubalus bubalis*.

A total number of 34,139 genes were retrieved from *Bubalus bubalis* from NCBI (30) for mapping. After manual curation, 34,067 genes (98.41%) were obtained, which were further used for mapping 129 mastitis associated genes. Of these, 101 genes were successfully mapped. Supplementary Table 1 shows details including the mapping location (chromosome coordinates), chromosome number, strand orientation, the total number of exons, gene type, organism based on the literature survey of these genes, whether the information was retrieved from cattle or buffalo studies along with references. Chromosome-wise mapping of all these 101 genes is depicted in Figure 2.

### Validation of Predicted TGP Genes in Water Buffalo Gene Expression Atlas

**Figure 3:**
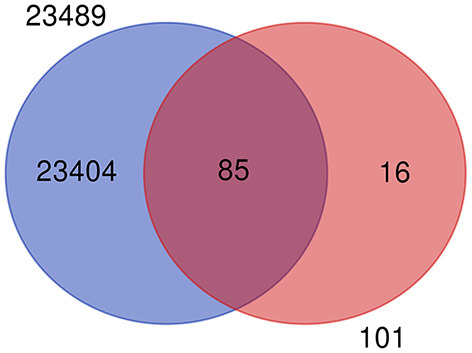
Venn diagram showing the common and unique genes when compared to gene expression atlas of water buffalo and genes from milk traits.

The mastitis associated genes under study are revalidated by using recently published work of the gene expression atlas (30) of the domestic water buffalo (*Bubalus bubalis*) containing 23,492 genes expressed across 50 different tissues. After removing the duplicates, the remaining 23,489 genes were used for the revalidation of 101 key candidate genes. It was observed that 85 out of 101 key candidate genes were common in 23,489 genes (Figure 3), except for genes that include: *selectin P (SELP), Amyloid beta precursor protein (APP), BTG anti-proliferation factor 1 (BTG1), lipopolysaccharide binding protein (LBP), CD46 molecule (CD46), lactotransferrin (LF), carboxypeptidase (CP), matrix metallopeptidase 9 (MMP9), BRCA1 DNA repair associated (BRCA1), RNA binding motif single stranded interacting protein 1 (RBMS1), ETS proto-oncogene 2, transcription factor (ETS2), transforming acidic coiled-coil containing protein 3 (TACC3), hepatocyte growth factor (HGF), serum amyloid A 3 (SAA3), orosomucoid 1 (ORM1)*, and *cytochrome c oxidase subunit II (COX2)*. The common gene list is included in Supplementary Table 2.

### Functional Prediction of Mastitis Associated Candidate Genes

The roles of the mastitis associated genes in ontological terms were explored by their functional annotation. An organism specific classification system for the functionally annotated genes using PANTHER (32) was obtained for *Bos taurus* only as it does not have *Bubalus bubalis*. A genetically close bovine species, *Bos taurus* was used for this analysis to predict the biological role of the genes. Out of 101 genes, 98 were annotated successfully on molecular, biological, and cellular functions based on their role and classification to a PANTHER family. The predicted protein of mastitis associated genes and their pathways were also obtained. For example, gene *enoyl-CoA hydratase and 3-hydroxyacyl CoA dehydrogenase (EHHADH)* are classified to PANTHER family, peroxisomal bifunctional enzyme (Family ID: PTHR23309). There are a total of 121 genes in this particular family. The molecular function of oxidoreductase activity and biological process includes fatty acid catabolic process and oxidation-reduction process. The protein class annotated for gene *EHHADH* is dehydrogenase. Supplementary Table 3 includes details of all the 98 annotated genes in *Bos taurus*.

Gene annotation with species *Bubalus bubalis* using DAVID revealed annotation of 26 genes (Table 1). With respect to biological processes, genes such as *toll like receptor 2 and 4 (TLR2 and TLR4), C-C motif chemokine ligand 3 (CCL3), complement C3 (C3), C-X-C motif chemokine ligand 8 (CXCL8)*, and *CD14 molecule (CD14)* were found to be involved in inflammatory response (44–47). These genes along with *S100 calcium binding protein A8 (S100A8)* and *lipopolysaccharide binding protein (LBP)* were involved in innate immune response (48, 49). *Interleukin1 beta (IL1B)* was involved in processes like fever generation, immune response, and positive regulation of cell division which provides evidence for the cause of symptoms in mastitis disease condition (50).

**Table 1:** Gene ontology analysis of candidate genes through DAVID.

**Description**	***P*-value**	**Genes**	**Fold enrichment**	**FDR**
**Cellular component**				
Extracellular space	0.0002	*LTF, IL1B, LPO, CCL3, C3, IL12B, CXCL8, LBP, IL6*	4.22	0.12
Cytokine	0.0159	*IL1B, CCL3, IL12B, CXCL8, IL6*	4.46	14.48
Signal	0.0227	*IL6, CD14, LPO, CSF2, C3, IL12B, TLR4, TLR2, CCL3, LY96, CXCL8, LBP, IL6*	1.66	20.07
Glycoprotein	0.0905	*CCL3, CD14, LPO, IL12B, IL6*	2.68	60.35
Secreted	0.1675	*CD14, IL1B, LPO, LY96, IL12B, TLR4, CXCL8, IL6*	1.74	83.29
Disulfide bond	0.2239	*IL6, CCL3, CD14, LPO, CSF2, IL12B, CXCL8, IL6*	1.43	91.57
**Biological process**				
Immune response	0.3433	*IL1B, C3, CXCL8, IL6*	1.83	99.03
Inflammatory response	0.0095	*IL6, CCL3, LY96, IL12B, CXCL8, TLR2*	3.77	10.03
Innate immune response	0.0392	*IL6, S100AB, CCL3, TLR2, LBP*	3.35	35.68
Leucine-rich repeat	0.0439	*IL6, NOD2, CCL3, TLR2*	4.58	39.17
Inflammatory response	0.0567	*IL6, CCL3, IL1B, TLR2*	4.12	43.42
Immunity	0.0963	*IL6, CCL3, CD14, TLR2*	3.35	62.78
Innate immunity	0.2356	*IL6, CCL3, TLR2*	3.09	92.73
Membrane	0.9985	*IL6, CCL3, TLR2*	0.40	100.00
**Molecular function**				
ATP-binding	0.2356	*NOD2, AKT3, ACTB*	3.09	92.73
Nucleotide-binding	0.2913	*NOD2, AKT3, ACTB*	2.68	96.52
ATP binding	0.4803	*NOD2, AKT3, ACTB*	1.82	99.60

The molecular functions in which most of these genes are involved are related to binding and kinase activity. For instance, *AKT serine/threonine kinase 3 (AKT3) and CCL3* are involved in kinase and ATP binding function (51, 52). *TLR2* and *TLR4* molecules are involved in transmembrane signaling receptor activity (53). Many genes are involved in calcium, metal, zinc, ion binding functions like *lactoperoxidase* (*LPO*), *calmodulin 2* (*CALM2*), *S100 calcium binding protein A8 (S100A8)*, in calcium-ion binding, *lactotransferrin (LTF)* in iron ion binding, and *tumor protein p53 (TP53)* in the metal-ion binding process (54–56).

Cellular processes include signal components, secreted molecules, glycoproteins, disulfide bond molecules, cytokines, and extracellular space. Maximum genes are involved in signaling processes, a list of genes is given in Table 1. *Interleukin 6 and 12B (IL6 and IL12B)* are involved in all the cellular processes as stated (57, 58). Protein family annotation for these 26 genes and information from protein databases such as Simple Modular Architecture Research Tool (SMART), Protein Information Resource (PIR), and InterPro is included with protein family ID and functions associated in *WBMSTDb* (59–61). The details are available at http://webtom.cabgrid.res.in/wbmstdb/index.php under the tab: “Search—Mastitis Gene Panel—Gene Annotation.”

The pathway analyses of these genes as identified through KEGG Mapper revealed that out of the 101 mastitis associated genes under study, there were six genes for which KEGG ontology was not assigned. These are, *mannose-binding protein A (MBLA), actin beta (ACTB), FEZ family zinc finger 2 (FEZF2), osteoclast stimulating factor 1 (OSTF1), RNA binding motif single stranded interacting protein 1 (RBMS1), and RELA proto-oncogene, NF-kB subunit (RELA)*.

A total of 225 pathways in various categories were classified, namely, metabolism, genetic and environmental information processing, cellular process, organismal systems such as immune system, and human diseases. The pathways revealed the involvement of these genes in diverse roles. The majority of these genes were involved in infection or disease susceptibility by microbial organisms like *Salmonella, Shigellosis, Pertussis, Legionellosis, Staphylococcus aureus, Yersinia*, etc. (Table 2).

**Table 2:** Genes of mastitis involved in various infectious pathways.

**Pathway**	**No. of genes**	**Genes involved**
*Helicobacter pylori* infection	2	*CXCL8, CCL5*
Pathogenic *Escherichia coli* infection	7	*BAX, FOS, ILIB, TIRAP, IL6, CXCL8, TLR4*
Salmonella infection	12	*BAX, FOS, MAP2K7, AKT3, IL1B, LY96, TIRAP, IL6, CXCL8, TLR2, TLR4, BIRC3*
Shigellosis	11	*BAX, C3, TP53, AKT3, IL1B, CSF2, PLCE1, CXCL8, TLR4, CCL5, BCL2*
Yersinia infection	7	*FOS, MAP2K7, AKT3, IL1B, IL6, CXCL8, TLR4*
Pertussis	12	*CALM2, C4A, C3, FOS, IL1B, LY96, TIRAP, IL6, IL12B, IRF1, CXCL8, TLR4*
Legionellosis	9	*HSPA8, C3, IL1B, IL6, IL12B, CXCLS, TLR2, TLR4, BCL2*
*Staphylococcus aureus* infection	5	*CFB, C4A, C3, C5AR1, SELP*
Tuberculosis	12	*BAX, CALM2, C3, AKT3, IL1B, LBP, TIRAP, IL6, IL12B, TLR2, TLR4, NOD2*
Human T-cell leukemia virus 1 infection	7	*BAX, FOS, TP53, AKT3, IL6, CSF2, RELB*
Human immunodeficiency virus 1 infection	8	*BAX, CALM2, CCR5, FOS, MAP2K7, AKT3, TLR2, TLR4*
Measles	11	*BAX, HSPA8, FOS, TP53, AKT3, IL1B, STAT3, IL6, IL12B, TLR2, TLR4*
Influenza A	8	*BAX, AKT3, IL1B, IL6, IL12B, CXCL8, TLR4, CCL5*
Hepatitis B	11	*BAX, FOS, MAP2K7, TP53, AKT3, STAT3, TIRAP, IL6, CXCL8, TLR2, TLR4*
Hepatitis C	4	*BAX, TP53, AKT3, STAT3*
Herpes simplex virus 1 infection	10	*BAX, C3, TP53, AKT3, IL1B, IL6, IL12B, TLR2, CCL5, BIRC3*
Human cytomegalovirus infection	12	*BAX, CALM2, CCR5, TP53, AKT3, IL1B, STAT3, IL6, CCL3, CXCL8, PTGS2, CCL5*
Kaposi sarcoma-associated herpesvirus infection	14	*BAX, CALM2, C3, CCR5, FOS, MAP2K7, TP53, AKT3, STAT3, IL6, CSF2, CXCL8, PTGS2, FGF2*
Epstein-Barr virus infection	9	*BAX, GADD45B, MAP2K7, TP53, AKT3, STAT3, IL6, RELB, TLR2*
Human papillomavirus infection	6	*BAX, TP53, AKT3, SPP1, IRF1, PTGS2*
Amoebiasis	7	*IL1B, IL6, IL12B, CSF2, CXCL8, TLR2, TLR4*
Malaria	6	*IL1B, IL6, SELP, CXCL8, TLR2, TLR4*
Toxoplasmosis	10	*ALOX5, HSPA8, CCR5, AKT3, STAT3, LY96, IL12B, TLR2, TLR4, BIRC3*
Leishmaniasis	8	*C3, FOS, IL1B, IL12B, NCF4, TLR2, TLR4, PTGS2*
Chagas disease (American trypanosomiasis)	11	*C3, FOS, AKT3, IL1B, IL6, CCL3, IL12B, CXCL8, TLR2, TLR4, CCL5*
African trypanosomiasis	3	*IL1B, IL6, IL12B*

Nine genes were found to be involved in apoptosis activity like *BCL2 associated X, apoptosis regulator (BAX), BCL2 related protein A1 (BCL2A1), MCL1 apoptosis regulator, BCL2 family member (MCL1), Fos proto-oncogene, AP-1 transcription factor subunit (FOS), growth arrest and DNA damage inducible beta (GADD45B), tumor protein p53 (TP53), AKT serine/threonine kinase 3 (AKT3), baculoviral IAP repeat containing 3 (BIRC3)*, and *mitogen-activated protein kinase kinase 7 (MAP2K7)*. Table 2 describes the mastitis associated genes involved in various infectious pathways.

### Prediction of Cross-Talking Proteins by Protein-Protein Network Analysis

The panels of 111 proteins were used for PPI network analysis. Among these, 10 proteins were buffalo specific, which were obtained directly from NCBI. The remaining 101 proteins were retrieved from the STRING database using their genetic codes. The construction of the PPI network revealed 83 nodes and 227 edges. These predicted proteins were further searched for other cross talking proteins in *Bubalus bubalis*. This resulted in a final PPI network of 193 nodes and 1,480 edges (Table 3). PPI predicted 1,707 proteins associated with mastitis disease in water buffalo. Based on the degree, between-ness, and closeness of the network, they were classified into two groups, *viz*., (1) directly interacting 83 proteins with cross-talk with 227 proteins as first-degree interaction (2) other predicted proteins (193) which are having cross-talks with 1,480 predicted proteins as second-degree interaction in the network.

**Table 3:** Network statistics of protein-protein interaction.

**Network parameters**	**First interacting partners**	**Other interacting partners**
Nodes	83	193
Edges	227	1480
Average node degree	5.47	15.3
Avg. local clustering coefficient	0.416	0.55
Expected number of edges	62	485
PPI enrichment *p*-value	<1.0e-16	<1.0e-16

**Figure 4:**
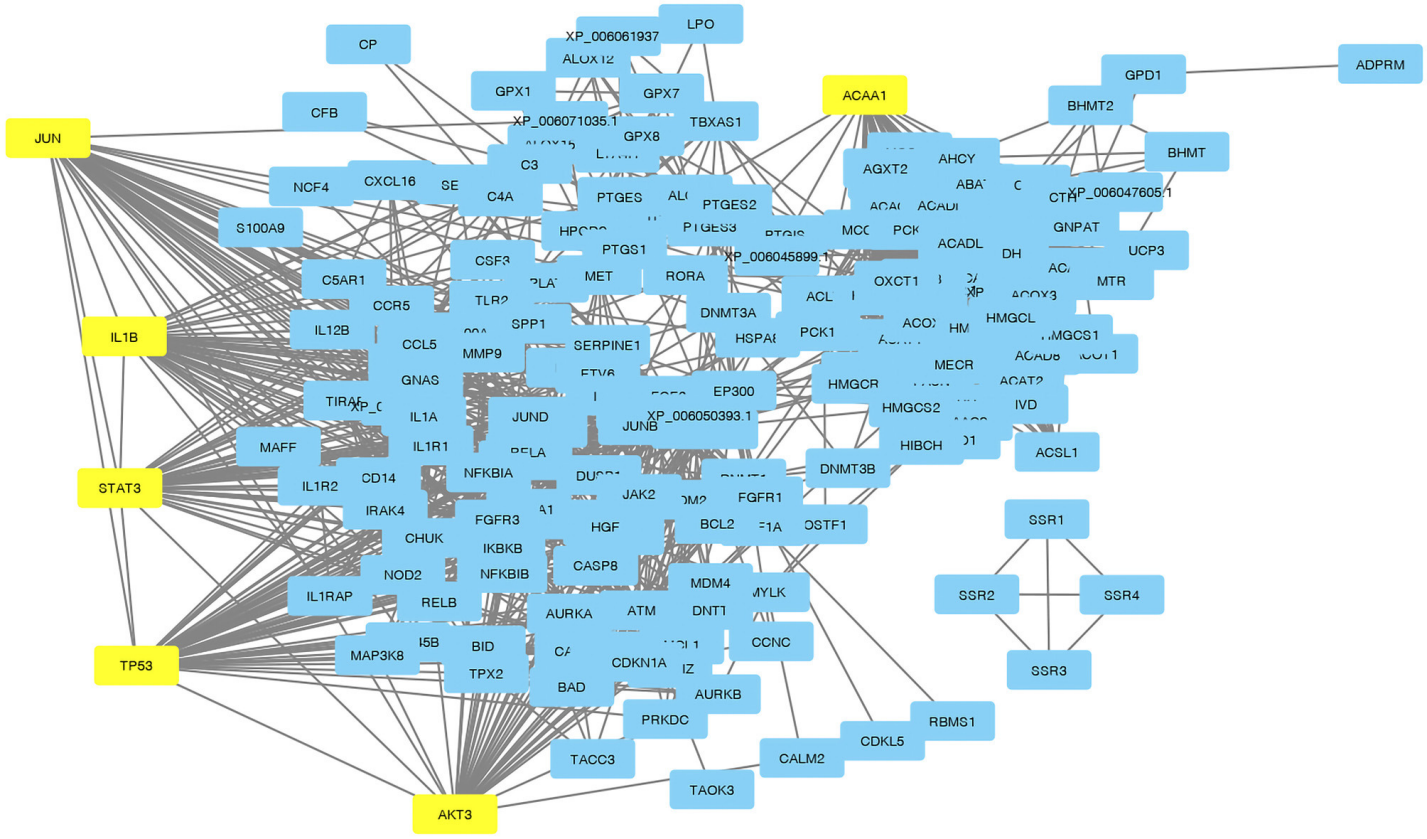
Protein-protein interactions with hub genes highlighted in yellow.

**Figure 5:**
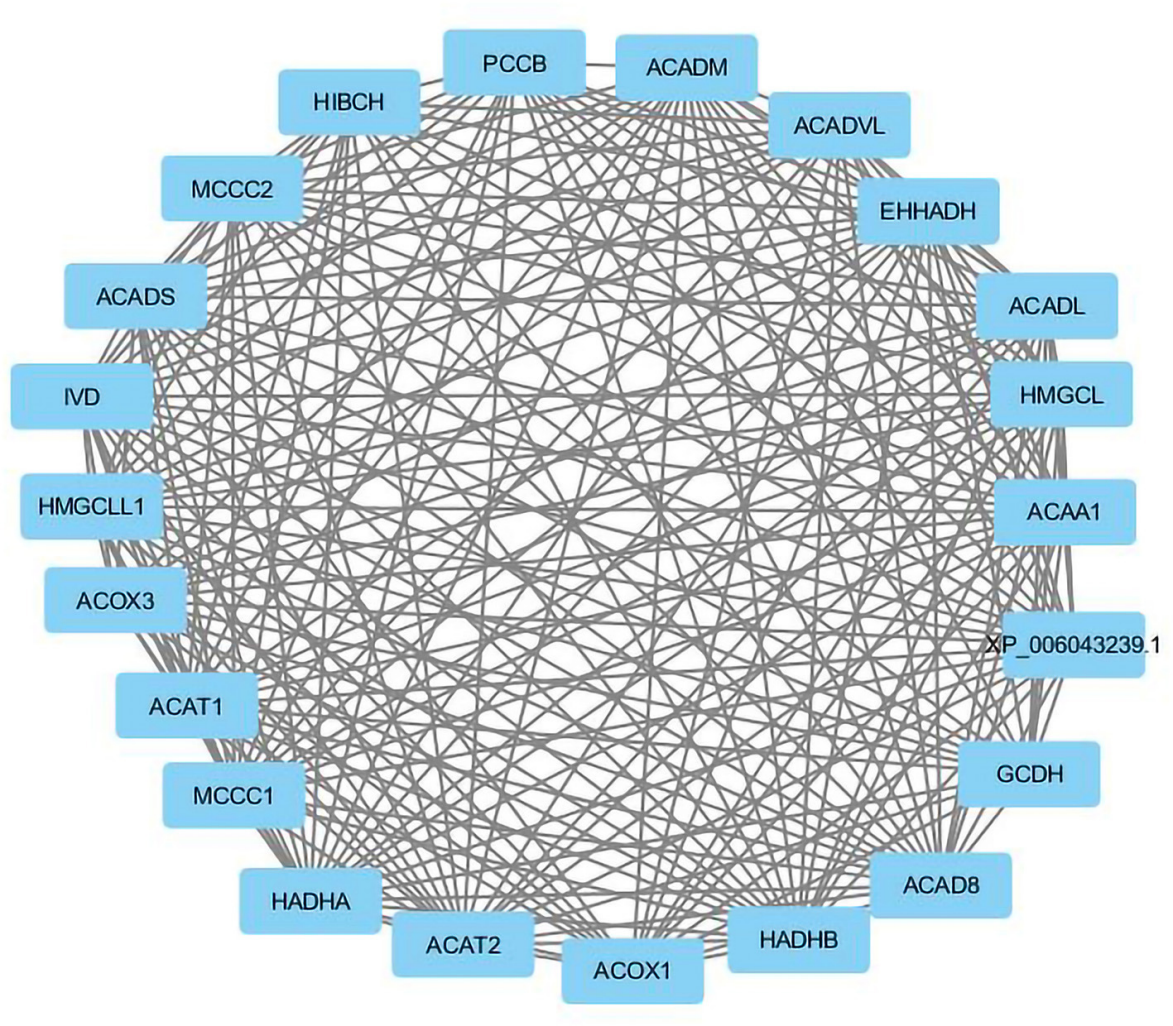
The cluster of proteins highly connected in protein-protein interaction network.

Visualization of a network with highly connected nodes using Cytoscape detected the hub proteins (Figure 4). Hub molecule analysis revealed six protein nodes (>40 edges) that are highly connected, *namely, TP53* (number of edges: 56), *Signal transducer and activator of transcription 3 (STAT3)* (number of edges: 49), *IL1B* (number of edges: 46), *Jun oncogene (JUN)* (number of edges: 45), *AKT3* (number of edges: 41), and *acetyl-CoA acyltransferase 1 (ACAA1)* (number of edges: 40). Out of these, *TP53, STAT3, IL1B*, and *AKT3* were found to be the first interacting partners, i.e., key candidate genes involved in mastitis, while *JUN* and *ACAA1* were the other interacting partners. Furthermore, using Cytoscape plugins, *CentiScape*, and *MCODE*, the highest PPI scores by three centrality methods were computed. These scores along with other parameters of these genes are shown in Table 4. A total of nine clusters identified from the PPI network were highly connected subnetworks. A cluster of highly connected proteins' sub-network revealed 22 nodes and 211 edges with a MCODE score value of 20 (Figure 5).

**Table 4:** List of genes showing PPI scores >10 identified through MCODE and CentiScape.

**Gene name**	**Score**	**Degree**	**Betweeness**	**Closeness**
*ACAA2*	16.1739	38	94.5830	0.00231
*ACAA1*	16.1739	40	1590.0820	0.00257
*ACADM*	15.4545	37	173.4419	0.00230
*MCCC1*	15.3874	35	191.9848	0.00229
*EHHADH*	15.2138	39	700.0301	0.00246
*HADHA*	15.2138	36	117.0222	0.00223
*ACAD8*	15.2138	30	63.9670	0.00224
*ACADL*	15.1858	33	80.3648	0.00218
*GCDH*	15.1579	21	6.3206	0.00204
*IVD*	14.9674	30	42.3387	0.00219
*PCCB*	14.6147	36	421.9345	0.00234
*HMGCL*	14.5067	35	123.8824	0.00228
*HMGCLL1*	14.5067	35	123.8824	0.00228
*ACOX3*	14.4118	25	69.0563	0.00220
*ACOX1*	14.4118	38	2221.2560	0.00271
*MCCC2*	14.0870	32	76.7229	0.00226
*HADHB*	13.9355	39	109.0790	0.00234
*HIBCH*	13.5882	23	528.4855	0.00226
*ACAT1*	13.5665	34	60.6734	0.00228
*ACAT2*	13.5665	34	60.6734	0.00228
*ACADS*	13.4933	29	45.8871	0.00213
*ACADVL*	13.0850	29	65.4278	0.00224
*ACADSB*	12.7717	27	35.4102	0.00212
*ABAT*	12.4265	22	405.8688	0.00219
*ACLY*	12.0652	39	3099.4708	0.00279
*ACSL1*	12.0000	16	5.6126	0.00207
*HIBADH*	11.8105	25	71.9954	0.00201
*HMGCR*	11.7363	20	211.7744	0.00242
*CS/Citrate Synthase Mitochondrial*	11.6769	39	1462.1305	0.00240
OXCT1	11.5652	24	25.5766	0.00216
MECR	11.5429	16	50.0551	0.00208
HMGCS2	11.3116	28	392.3604	0.00247
AACS	11.1176	23	39.1651	0.00219

### Evaluation of Mastitis TGP Based Variant Mining (SNPs and Indels)

The overall read mapping rate and concordant pair alignment rate of RNA-Seq data on the reference assembly was 94.4 and 88.9%, respectively. On mapping the genomic coordinates of SNPs and indels from the VCF file of mammary tissues transcriptome over coordinates of 101 genes using in-house Perl scripts, a total of 1,458 SNPs and indels were obtained. Out of these, 1,306 were SNPs and 152 were indels. The highest number of SNPs and indels (261) was observed for the gene, *BCL2* apoptosis regulator located on chromosome number 22 which is involved in apoptosis regulating activity. Gene *CD46* was mapped for 116 SNPs and indels. For the genes *MAF bZIP transcription factor F (MAFF), APP, signal sequence receptor subunit 1 (SSR1)*, and *CP*: 42, 37, 36, and 36 SNPs and indels were mapped. Similarly, for 11 genes, *namely, uncoupling protein 3 (UCP3), DNA nucleotidylexotransferase (DNTT), C-C motif chemokine ligand 20 (CCL20), FEZ family zinc finger 2 (FEZF2), colony stimulating factor 2 (CSF2), CCL3, interleukin 6 (IL6), Acyl CoA thioesterase 1 (ACOT1), SAA3, MBLA*, and *Orosomucoid 1 (AGP)*, there was no SNP and indel mapping. Supplementary Table 4 has the details of the SNP and indels mapped for the predicted mastitis genes.

### Development of Web Resource: Water Buffalo-MSTdb (*WBMSTDb*)

**Figure 6:**
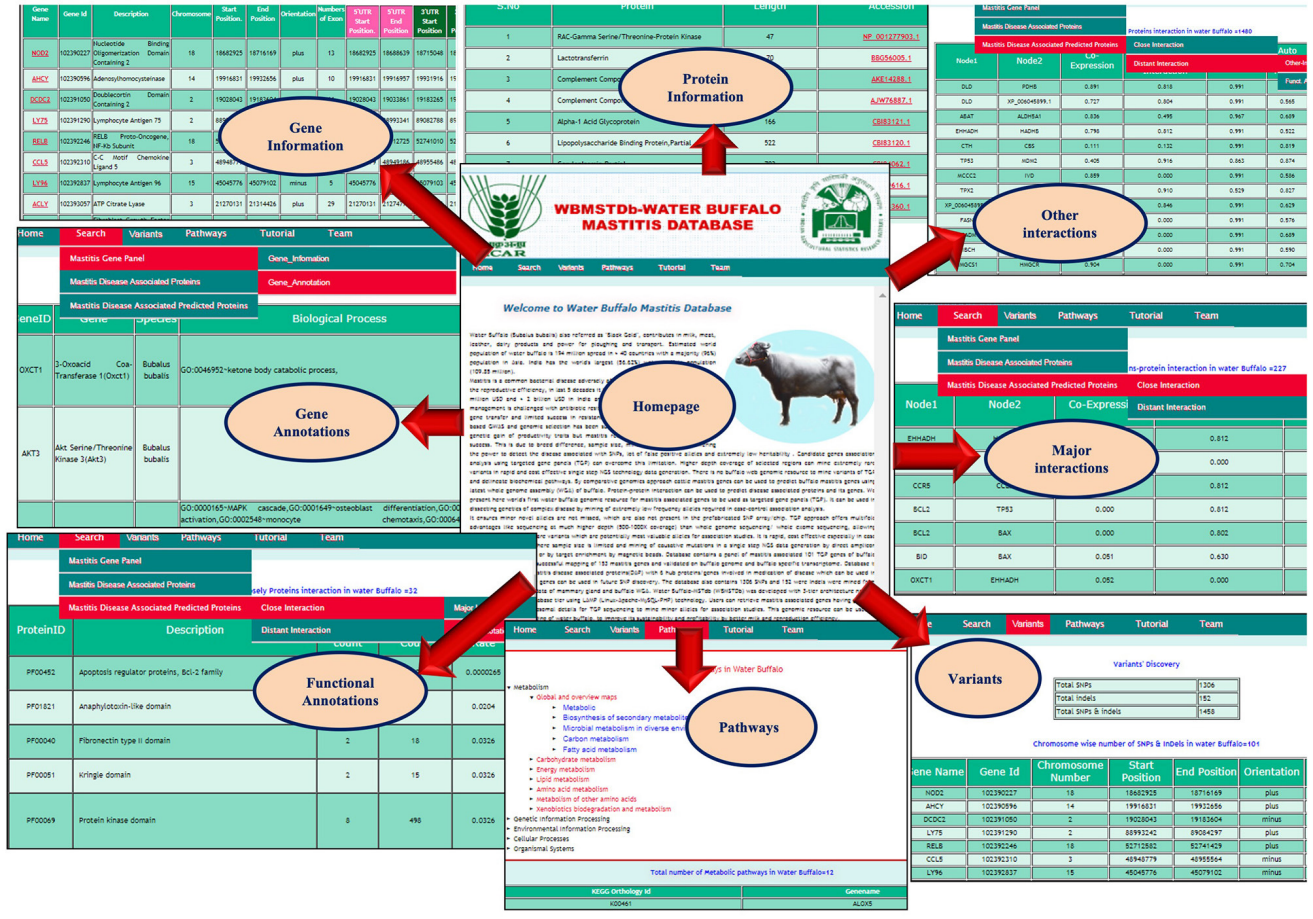
The interface of the WBMSTDb for various type of searches.

Water Buffalo Mastitis Database (*WBMSTDb*) is an open source and user-friendly web resource of targeted gene panels, which can be used for academic purposes in future mastitis association studies (website: http://webtom.cabgrid.res.in/wbmstdb/). WBMSTDb has five different tabs, *namely, Home, Search, Pathways, Tutorial*, and *Team*. *Home* tab deals with the brief introduction of this web resource along with various datasets related to mastitis cataloged in it. The objectives, developmental procedures, and rationale of its development are well-documented here. The *Search* tab is categorized into three sub-tabs, i.e., *Mastitis Gene Panel, Mastitis Disease Associated Proteins*, and *Mastitis Disease Associated Predicted Proteins*. Furthermore, the sub-tab *Mastitis Gene Panel* is categorized into two sub-sub-tabs, *namely*, “Gene Information” and “Gene Annotation.” The “Gene Information” sub-sub-tab has the information related to Gene symbol, Gene ID, Gene Description, Chromosome Number, Gene Start Position, Gene End position, Orientation, and Exon Counts. Provision has been made for the browser for simpler visualization. As users may have targeted regions within the gene for SNP mining, like UTR region or exonic regions only, so to cater to such needs, provision has been made for the convenient visualization of regions of each ORF by integrating the TGP with the genome browser. The “Gene Annotation Information” sub-sub tab has information related to Gene ID, Gene name, Species, GO term BP (Biological processes), GO term CC (Cellular components), GO term MF (Molecular Functions), Interpro database, the PIR superfamily database, SMART database, and Up keywords. The “*Mastitis Disease Associated Proteins*” sub-tab provides information on Protein names, Length, and accession Numbers. The “*Mastitis Disease Associated Predicted Proteins*” sub-tab provides two types of interaction images, i.e., “*Major Interaction*” and “*Distant Interaction*.” The information of PPI provides Node1, Node2, Neighborhood chromosome, Gene fusion, Phylogenetic co-occurrence, Homology, Co-expression, Experimentally determined interaction, Database annotation, and Auto-text mining. The “*Function Annotation*” provides ProteinID, Description, Gene-observed, Gene-background, False-discovery rate, Matched proteinID, Database. The “*Pathways*” tab deals with the information like Pathways name, Pathways Id, Numbers of Enzyme, Enzyme, Sequence of Enzyme count, Sequence Id, and Pathway Figures. Users can download the targeted gene panel for very high coverage (600–1000X) sequencing to mine for extremely low frequency alleles potentially associated with mastitis disease. The *Tutorials* tab contains the guidelines for users and terminology used in the database contents. Finally, the *Team* tab has the contact list of researchers involved in the analyses and development of the web resource, *WBMSTDb*. The various interfaces for users are shown systematically in Figure 6.

## Discussion

### Mining and Mapping of Bovine Mastitis Associated Genes

In a panel of successfully identified 129 mastitis associated genes, 110 and 19 genes were from cattle and buffalo populations, respectively. This availability of a higher number of mastitis genes of cattle in GenBank is expected as cattle are a much more widely distributed and well-studied bovine species than water buffalo. The successful mapping of mastitis associated genes (101) of heterologous species cattle over the water buffalo genome reveals their extensive conservation during the divergence of these bovine species. This high conservation agrees with earlier mapping studies of water buffalo depicting syntenic block coverage of >80% (62). Many mastitis associated genes like *complement C5 (C5)* and *chemokine (C-X-C motif) receptor 1 (CXCR1)*, are reported to be conserved as high as up to 99% sequence similarity (63, 64).

This extensive conservation of cattle genes in water buffalo is due to their common ancestor, homologous proteins, common gene family, common CNV, common gene statistics, across species selection signature of milk pathways are also revealed in buffalo genome Benchmarking Universal Single-Copy Orthologs (BUSCO) mapping studies (62). The remaining 28 cattle genes, which could not be mapped over the buffalo genome, might be due to their acquired species specificity in cattle during species divergence (Supplementary Table 5). This structural genomic difference of river buffalo and cattle has also been reported in comparative genomic and transcriptomic studies (65).

The key candidate genes are mapped to the chromosome location and position using the entire set of buffalo genes from NCBI. Maximum key genes of mastitis were mapped to chromosome 3 as also reported in water buffalo previous studies (66, 67). Interestingly, many alleles of mastitis genes of chromosome 3 are also associated with fertility traits. Mastitis also affects fertility, as this disease may lead to early embryonic death, infertility, or sterility in water buffaloes (68).

### Validation of Predicted TGP Genes in Water Buffalo Gene Expression Atlas

The successful mapping of 85% TGP genes over water buffalo species specific gene expression atlas demonstrates the reliability of predicted genes as they get transcribed in buffalo tissues. The remaining non-validated (15%) TGP genes might be due to spatiotemporal gene expression data in the atlas. In an atlas, all genes are not expected to express all the time. This will vary with the tissue as well as in the timeline for the sampling of transcriptome data generation. This spatiotemporal gene expression in the milk tissue of bovines has been reported (69).

### Functional Prediction of Mastitis Associated Candidate Genes

Mastitis genes regulating the proteins may have a significant functional role in the modulation and regulation of mastitis disease. Proteins like *complement C4 (C4), BRCA1, TLR4, MBLA*, and *calcium voltage-gated channel auxiliary subunit alpha2delta 1 (CACNA2D1)* are reported potential biomarkers in mastitis (70) which were also found in our study. The functional prediction of the key genes associated with mastitis disease was found to be involved in the immune and inflammatory response. For instance, genes like *CXCL8, CD14, S100A8, CSF2, C3, Interferon regulatory factor 1 (IRF1), IL1B, IL6, LPO, LBP, TLR2, and TLR4* are well-known immune and inflammatory responsive genes (71). Mastitis is a complex disease causing inflammation of the mammary gland where origin, severity, and outcome of the disease depend on the environment, pathogen virulence, and host immunity (9). Mastitis is characterized by physical, chemical, and biological changes in milk quality like the presence of blood, water, pus, clots, flakes, and shreds consisting of fibrin as well as cellular debris, udder enlargement, somatic cell count, and asymmetry of the gland (72). In our predictive functional annotation analysis, the key candidate genes were found to mediate such responses including mastitis fever generation (73).

### Prediction of Cross-Talking Proteins by Protein-Protein Network Analysis

A protein-protein interaction study has revealed the various cross talking proteins of mastitis genes in water buffalo that can be used to understand the disease and associated proteins (74). Mastitis associated predicted proteins can be classified using the degree, between-ness, and closeness of the network (75). The top hub molecules of the interaction network predicted were *TP53, STAT3, IL1B*, and *AKT3*. The functional characterization of these proteins provides evidence of their role in signaling and the symptoms caused by mastitis. For example, *AKT3* expression increases during lactation in the mammary gland and immune cells, is also involved in the *TLR* pathways regulating as proinflammatory cytokines (51). The role of *IL1B* as an inflammatory cytokine in mastitis disease resistance has been reported in water buffalo (76). *TP53* gene has been reported as a key candidate gene that drives mastitis resistance genes (77). Furthermore, *IL1B* is reported as a hub molecule in cattle mastitis (78). The *Serotransferrin (TF)* gene is reported as a major regulator of a set of genes that are responsible for the resistance/susceptibility of mastitis (79). Enhanced *semaphorin 5A (SEMA5A)* gene expression has been reported to induce at least nine genes related to the host's immune response including *tumor necrosis factor alpha (TNF-a) and interleukin 8 (IL-8)* (80). Since the heritability of adaptive immune traits ranges from 0.25 to 0.35 in dairy cattle, thus the genes associated with it are valuable in mastitis resistance breeding (81). The high immune responder approach has been utilized to assess the dairy cattle's ability to trigger antibody and cell-mediated immune response against pathogens. The cows with superior adaptive immune response have been reported to show a lower occurrence of mastitis and metritis (82). How these predicted proteins have cross talk with mastitis associated proteins needs to be studied further to understand the genetic mechanism of this disease by a bottom up approach, predicting additional genes from these putative proteins. The genes of enlisted DAP can be used for future SNP discovery as well as for designing drugs (83).

### Evaluation of Mastitis TGP Based Variant Mining (SNPs and Indels)

Variant mining for SNP (1,306) and indel (152) were found to be limited in number with respect to the total genes targeted (101). Since the genic region confines to <3% of the total genome, transcriptome data is expected to have less SNPs for WGS based SNP mining. In the case of water buffalo, the average exon length is 181.74 bp with respect to the average intron length of 3,435 bp, which also indicates a lower abundance of SNPs (62) in the genic region. This lower number of SNPs in genic region was also reported in water buffalo targeted sequencing in the Jafarabadi breed (84). In another study of the Murrah water buffalo breed, genic region SNPs (79.40%) were found in nearly one-fifth of non-genic region SNPs (19.26%) in terms of their relative abundance (85). Though exonic variations are very limited in number, they do play an important role in population differentiation at the genetic level (86). Non-synonymous SNP polymorphism in the genic region of mastitis genes is very valuable for mastitis disease resistance breeding in bovines (87).

Such genic region SNPs with known physical map locations have been used in dissecting mastitis resistance QTL in dairy cattle (15). The use of indel as a marker has the advantage of making genotyping and multiplexing easier (88). Such indel polymorphism in milk genes and its trait association studies are reported in goats (89). A higher number of variants, i.e., 261 and 116, were found in gene *BCL2* (Gene ID 281020) and *CD46* (Gene 280851), respectively. A longer gene is expected to have a higher number of variants which is also evident by the higher number of exons (*BCL2* with 7 and CD46 with 17 exons). *BCL2* is also known to have copy number variants (90) and isforms (91), which might have contributed to the increased number of SNPs. The SNP of the *BCL2* gene has been found to be associated with heat stress in water buffalo (92). Similarly, the SNP of gene *CD46* was also found to be associated with mastitis in bovines (93). SNP studies of TGP genes can pave the knowledge discovery of pathway genes mediating mastitis disease by integrative analysis with transcriptomic data (24, 94).

### Development of Web Resource: Water Buffalo-MSTdb (*WBMSTDb*)

The Water Buffalo-MSTdb (WBMSTDb) is the first targeted gene panel genomic resource of water buffalo for mastitis association studies. It is freely accessible to the global community. This web genomic resource can be used for the marker discovery required in mastitis resistance breeding of water buffalo. Users can download the complete ORF of the genes to be targeted for mining the variants. A genome browser can assist in the depiction of various parts of an ORF of the gene. Since TGP contains the entire open reading frame having 5′UTR, exons, introns, and 3′UTR with genomic coordinates, it can be used for SNP, simple-sequence repeat (SSR), and indel marker discovery, which are required in association studies for mastitis resistance breeding. Such marker discovery assay can be designed using NGS technology with the sequencing of very high depth coverage (500–1000X), by both target enrichment method (>50 gene target genes) using magnetic beads or direct amplicon sequencing (if the number of genes are <50) in a single rapid cost-effective assay (25).

Many mastitis candidate gene association studies have been successfully reported for cattle. For example, in case of *mannan binding lectin serine peptidase 2 (MASP2)* (95), *TLR4* (96), *ATPase Na*^+^*/K*^+^ *transporting subunit alpha 1 (ATP1A1)* (97), *IL-8* (98), *mitogen-activated protein kinase kinase kinase kinase 4* (*MAP4K4*) (99), lipocalin-2 *(LNC2*) (100), *TLR2* (101), *CD14* (102), *TLR4* (103), Exon3 *TL-4* (104), *CD46* (93), *phosphodiesterase 9A (PDE9A)* (105), and *insulin-like growth factor 1 (IGF-1)* (106) have been reported. A similar approach is required in water buffalo for mastitis resistance breeding. *Target gene panel* approach with very limited sample size and limited gene number with case-control association study model has been successful in the diagnosis of susceptible individuals. A target gene panel covering both coding and non-coding region having SSR has been successfully used for improved diagnostic of disease (107).

## Conclusion

The mapping of cattle mastitis associated genes in water buffalo genome assembly has revealed their extensive conservation. Mapping can be used advantageously in making targeted gene panels (TGP), which are required for low frequency variant mining in the water buffalo population. Such low frequency alleles are very valuable in disease association analysis and often get missed in GWAS. The limitations of GWAS studies in mastitis disease resistance breeding could be overcome by supplementing candidate gene association analysis approaches using our developed TGP in *WBMSTDb*. Protein-protein interaction has predicted disease associated protein and revealed the biochemical pathway mediating that takes place as a result of mastitis disease. These proteins can be used for future drug design. The predicted TGP genes were successfully validated in a transcriptome of various buffalo tissues. Successful variant mining was also done using the transcriptome data of the buffalo mammary gland. This TGP can be used in buffalo breeding programs for selection and culling and better milk and reproductive efficiency.

## Data Availability Statement

All datasets generated for this study are included in the web resource: http://webtom.cabgrid.res.in/wbmstdb/.

## Author Contributions

DK conceived the theme of the study. SJ, JJ, MI, JK, AG, and UA performed the computational analysis and developed genomic resources. SJ, JJ, MI, and DK drafted the manuscript. VN, SK, RK, TD, AR, and DK edited the manuscript. All co-authors read and approved the final manuscript.

## Conflict of Interest

The authors declare that the research was conducted in the absence of any commercial or financial relationships that could be construed as a potential conflict of interest.
