# Supplementary material for: Genome Wide Prediction, Mapping and Development of Genomic Resources of Mastitis Associated Genes in Water Buffalo
Source: Front Vet Sci. 2021 Jun 18;8:593871. doi: 10.3389/fvets.2021.593871 (PMC8253262; doi:10.3389/fvets.2021.593871)
Supplement: Supplementary file 1 [file Table_1.docx]

**Genome wide prediction, mapping and development of genomic resources of mastitis associated genes in water buffalo**

Sarika Jaiswal^1^, Jaisri Jagannadham^1^, Juli Kumari^1^, Mir Asif Iquebal^1^, Anoop Kishor Singh Gurjar^1^, Varij Nayan^2^, UB Angadi^1^, Sunil Kumar^1^, Rakesh Kumar^3^, TK Datta^3^, Anil Rai^1^, Dinesh Kumar^1*^

^1^Centre for Agricultural Bioinformatics, ICAR-Indian Agricultural Statistics Research Institute, New Delhi-110012

^2^ICAR-Central Institute for Research on Buffaloes, Hisar, Haryana 125001

^3^Animal Biotechnology Centre, ICAR-National Dairy research Institute, Karnal, Haryana 132001

*****Corresponding Author: dinesh.kumar@icar.gov.in

**Supplementary Table 1**. Description of mastitis associated genes along with chromosome location, position, strand orientation, number of exons, gene-type, and organism reported from literature study and reference. Abbreviation PC: Protein Coding.

| Gene Symbol | Description | Chromosome | Start position on chromosome | End position on chromosome | Orientation | Exon count | Gene Type | Organism | Reference |
| --- | --- | --- | --- | --- | --- | --- | --- | --- | --- |
| ACLY | ATP citrate lyase | 3 | 21270131 | 21314426 | plus | 29 | PC | Cattle | 107, 108 |
| ACOT1 | acyl-coenzyme A thioesterase 1 | 11 | 18256506 | 18271647 | minus | 3 | PC | Cattle | 108 |
| ACSL1 | acyl-CoA synthetase long chain family member 1 | 1 | 30341810 | 30407173 | plus | 23 | PC | Cattle | 108 |
| ACTB | actin beta | 24 | 3757940 | 3761410 | minus | 6 | PC | Cattle | 107, 109 |
| AGP | orosomucoid 1 | 3 | 166216968 | 166219861 | plus | 5 | PC | Cattle | 107, 109 |
| AHCY | adenosylhomocysteinase | 14 | 19916831 | 19932656 | plus | 10 | PC | Cattle | 110 |
| AKT3 | AKT serine/threonine kinase 3 | 5 | 47554527 | 47837281 | minus | 17 | PC | Cattle | 50 |
| ALOX5 | arachidonate 5-lipoxygenase | 4 | 163691453 | 163738329 | minus | 14 | PC | Cattle | 111 |
| APP | amyloid beta precursor protein | 1 | 54928393 | 55236109 | plus | 18 | PC | Cattle | 112 |
| B2M | beta-2-microglobulin | 11 | 158245 | 164476 | minus | 4 | PC | Cattle | 113 |
| BAX | BCL2 associated X, apoptosis regulator | 18 | 55540482 | 55544744 | plus | 6 | PC | Cattle | 114 |
| BCL2 | BCL2 apoptosis regulator | 22 | 590147 | 785693 | plus | 7 | PC | Cattle | 108 |
| BCL2A1 | BCL2 related protein A1 | 20 | 43458172 | 43470928 | plus | 3 | PC | Cattle | 114 |
| BFSP1 | beaded filament structural protein 1 | 14 | 45947181 | 45986083 | plus | 8 | PC | Cattle | 112 |
| BID | BH3 interacting domain death agonist | 4 | 11044202 | 11062286 | plus | 8 | PC | Cattle | 108 |
| BIRC3 | baculoviral IAP repeat containing 3 | 16 | 78623153 | 78641369 | plus | 11 | PC | Cattle | 108 |
| BRCA1 | BRCA1 DNA repair associated | 3 | 20209334 | 20286775 | plus | 26 | PC | Buffalo; Cattle | 115, 116 |
| BTG1 | BTG anti-proliferation factor 1 | 4 | 98236506 | 98239213 | plus | 2 | PC | Cattle | 108 |
| C3 | complement C3 | 9 | 93485444 | 93521626 | minus | 41 | PC | Buffalo; Cattle | 62 |
| C4A | complement C4 | 2 | 25590016 | 25604860 | plus | 41 | PC | Cattle | `117 |
| C5AR1 | complement C5a receptor 1 | 18 | 54314694 | 54329591 | plus | 2 | PC | Buffalo; Cattle | 28, 62,107, 119,120 |
| CACNA2D1 | calcium voltage-gated channel auxiliary subunit alpha2delta 1 | 8 | 37921326 | 38447975 | plus | 44 | PC | Cattle | 118 |
| CALM2 | calmodulin 2 | 12 | 29261756 | 29274718 | minus | 6 | PC | Cattle | 108 |
| CCL20 | C-C motif chemokine ligand 20 | 2 | 168974704 | 168978216 | plus | 4 | PC | Cattle | 108 |
| CCL3 | C-C motif chemokine ligand 3 | 3 | 49105120 | 49106558 | minus | 3 | PC | Cattle | 108 |
| CCL5 | C-C motif chemokine ligand 5 | 3 | 48948779 | 48955564 | minus | 3 | PC | Cattle | 113 |
| CCR5 | C-C motif chemokine receptor 5 | 21 | 53090244 | 53114601 | minus | 5 | PC | Cattle | 121 |
| CD14 | CD14 molecule | 9 | 59472581 | 59475345 | plus | 2 | PC | Cattle | 28,107,119,120,122 |
| CD46 | CD46 molecule | 5 | 5401219 | 5439815 | plus | 16 | PC | Cattle | 92 |
| CDK8 | cyclin dependent kinase 8 | 13 | 57597995 | 57670492 | plus | 14 | PC | Cattle | 110 |
| CDKL5 | cyclin dependent kinase like 5 | X | 12421285 | 12596766 | plus | 23 | PC | Cattle | 108 |
| CEBPB | CCAAT enhancer binding protein beta | 14 | 5292987 | 5295000 | minus | 1 | PC | Cattle | 108 |
| CFB | complement factor B | 2 | 25555077 | 25561115 | plus | 18 | PC | Cattle | 108 |
| CP | ceruloplasmin | 1 | 163501846 | 163562346 | plus | 22 | PC | Cattle | 123 |
| CSF2 | colony stimulating factor 2 | 9 | 88897909 | 88899933 | plus | 4 | PC | Cattle | 107 |
| CSF3 | colony stimulating factor 3 | 3 | 23040727 | 23043152 | minus | 5 | PC | Cattle | 107 |
| CXCL16 | C-X-C motif chemokine ligand 16 | 3 | 36771027 | 36774569 | minus | 5 | PC | Cattle | 108 |
| CXCL8 | C-X-C motif chemokine ligand 8 | 7 | 28769703 | 28772345 | minus | 4 | PC | Buffalo; Cattle | 28, 107, 119,120 |
| DCDC2 | doublecortin domain containing 2 | 2 | 19028043 | 19183604 | minus | 11 | PC | Cattle | 113 |
| DNTT | DNA nucleotidylexotransferase | 23 | 17436567 | 17469020 | plus | 11 | PC | Cattle | 112 |
| DUSP1 | dual specificity phosphatase 1 | 19 | 4326581 | 4329684 | minus | 4 | PC | Cattle | 108 |
| EHHADH | enoyl-CoA hydratase and 3-hydroxyacyl CoA dehydrogenase | 1 | 126702951 | 126756081 | plus | 7 | PC | Cattle | 108 |
| ETS2 | ETS proto-oncogene 2, transcription factor | 1 | 195766624 | 195786350 | plus | 10 | PC | Cattle | 28, 107 |
| ETV6 | ETS variant transcription factor 6 | 4 | 21927676 | 22217154 | plus | 9 | PC | Cattle | 108 |
| FEZF2 | FEZ family zinc finger 2 | 21 | 38980959 | 38985831 | plus | 5 | PC | Cattle | 28,79, 107 |
| FGF2 | fibroblast growth factor 2 | 17 | 38184720 | 38239832 | plus | 3 | PC | Cattle | 124 |
| FOS | Fos proto-oncogene, AP-1 transcription factor subunit | 11 | 16812238 | 16815530 | minus | 4 | PC | Cattle | 108 |
| GADD45B | growth arrest and DNA damage inducible beta | 9 | 90203976 | 90206128 | plus | 4 | PC | Cattle | 108 |
| GNAS | guanine nucleotide-binding protein G(s) subunit alpha isoforms short | 14 | 26056556 | 26110548 | plus | 15 | PC | Cattle | 125 |
| GPD1 | glycerol-3-phosphate dehydrogenase 1 | 4 | 90491307 | 90497688 | plus | 9 | PC | Cattle | 108 |
| HADHB | hydroxyacyl-CoA dehydrogenase trifunctional multienzyme complex subunit beta | 12 | 72905983 | 72937636 | minus | 17 | PC | Cattle | 108 |
| HGF | hepatocyte growth factor | 8 | 38707241 | 38792681 | plus | 22 | PC | Cattle | 108 |
| HP | haptoglobin | 18 | 38758639 | 38764791 | minus | 7 | PC | Cattle | 108 |
| HSPA8 | heat shock protein family A (Hsp70) member 8 | 16 | 51229014 | 51233506 | plus | 9 | PC | Cattle | 108 |
| IL12B | interleukin 12B | 9 | 39936556 | 39955232 | plus | 11 | PC | Cattle | 107 |
| IL1B | interleukin 1 beta | 12 | 46284574 | 46293003 | minus | 7 | PC | Buffalo; Cattle | 28, 107, 113, 119,120 |
| IL6 | interleukin 6 | 8 | 31215270 | 31219246 | plus | 5 | PC | Cattle | 28, 107, 113 |
| IRF1 | interferon regulatory factor 1 | 9 | 89358528 | 89365523 | minus | 10 | PC | Cattle | 108 |
| LBP | lipopolysaccharide binding protein | 14 | 16357503 | 16392221 | minus | 15 | PC | Cattle | 28, 107, 119,120 |
| LPO | lactoperoxidase | 3 | 54208872 | 54232393 | minus | 12 | PC | Cattle | 108 |
| LTF | lactotransferrin | 21 | 53019196 | 53052145 | plus | 17 | PC | Buffalo; Cattle | 28,33,107, 111,126 |
| LY75 | lymphocyte antigen 75 | 2 | 88993242 | 89084297 | plus | 35 | PC | Cattle | 108 |
| LY96 | lymphocyte antigen 96 | 15 | 45045776 | 45079102 | minus | 5 | PC | Cattle | 108 |
| MAFF | MAF bZIP transcription factor F | 4 | 10229250 | 10238406 | minus | 3 | PC | Cattle | 108 |
| MAP2K7 | mitogen-activated protein kinase kinase 7 | 9 | 94605470 | 94615280 | minus | 12 | PC | Cattle | 108 |
| MAP3K8 | mitogen-activated protein kinase kinase kinase 8 | 14 | 48499556 | 48526611 | minus | 9 | PC | Cattle | 108 |
| MBLA | mannose-binding protein A | 4 | 154976531 | 154981859 | minus | 4 | PC | Cattle | 92 |
| MCL1 | MCL1 apoptosis regulator, BCL2 family member | 6 | 19848966 | 19853726 | plus | 3 | PC | Cattle | 108 |
| MMP9 | matrix metallopeptidase 9 | 14 | 8765042 | 8772373 | minus | 13 | PC | Cattle | 114 |
| NCF4 | neutrophil cytosolic factor 4 | 4 | 44932702 | 44951343 | minus | 4 | PC | Cattle | 127 |
| NFKBIZ | NFKB inhibitor zeta | 1 | 90942949 | 90974380 | plus | 14 | PC | Cattle | 108 |
| NOD2 | nucleotide binding oligomerization domain containing 2 | 18 | 18682925 | 18716169 | plus | 13 | PC | Cattle | 128 |
| OSTF1 | osteoclast stimulating factor 1 | 3 | 113965232 | 114025684 | plus | 11 | PC | Cattle | 110 |
| OXCT1 | 3-oxoacid CoA-transferase 1 | 19 | 32391695 | 32559174 | plus | 17 | PC | Cattle | 108 |
| PLAT | plasminogen activator, tissue type | 1 | 8520535 | 8544648 | plus | 14 | PC | Cattle | 113 |
| PLCE1 | phospholipase C epsilon 1 | 23 | 15203933 | 15572597 | plus | 33 | PC | Cattle | 110 |
| PRKDC | protein kinase, DNA-activated, catalytic subunit | 15 | 62849792 | 62976253 | plus | 87 | PC | Cattle | 110 |
| PTGS1 | prostaglandin-endoperoxide synthase 1 | 12 | 92804414 | 92829321 | plus | 12 | PC | Cattle | 28, 107, 111 |
| PTGS2 | prostaglandin-endoperoxide synthase 2 | 5 | 13291950 | 13299493 | plus | 10 | PC | Cattle | 111 |
| RBMS1 | RNA binding motif single stranded interacting protein 1 | 2 | 88371004 | 88617706 | plus | 18 | PC | Cattle | 108 |
| RELA | RELA proto-oncogene, NF-kB subunit | 5 | 120420240 | 120429172 | minus | 11 | PC | Cattle | 108 |
| RELB | RELB proto-oncogene, NF-kB subunit | 18 | 52712582 | 52741429 | plus | 13 | PC | Cattle | 108 |
| RORA | RAR related orphan receptor A | 11 | 53639029 | 54450465 | minus | 15 | PC | Cattle | 28, 107, 110 |
| S100A8 | S100 calcium binding protein A8 | 6 | 16790208 | 16791292 | plus | 3 | PC | Cattle | 108 |
| SAA1 | serum amyloid A protein-like | 5 | 102085523 | 102088779 | minus | 4 | PC | Cattle | 108 |
| SAA3 | serum amyloid A protein-like | 5 | 102063403 | 102067260 | minus | 4 | PC | Cattle | 28, 108 |
| SELP | selectin P | 5 | 43841921 | 43884226 | plus | 17 | PC | Cattle | 108 |
| SLC18A2 | solute carrier family 18 member A2 | 23 | 36989210 | 37030313 | plus | 16 | PC | Cattle | 112 |
| SPP1 | secreted phosphoprotein 1 | 7 | 80782654 | 80789613 | plus | 7 | PC | Cattle | 112 |
| SSR1 | signal sequence receptor subunit 1 | 2 | 4439705 | 4457888 | minus | 9 | PC | Cattle | 110 |
| STAT3 | signal transducer and activator of transcription 3 | 3 | 20869046 | 20943851 | plus | 24 | PC | Cattle | 108 |
| TACC3 | transforming acidic coiled-coil containing protein 3 | 7 | 950764 | 961155 | plus | 18 | PC | Cattle | 108 |
| TAOK3 | TAO kinase 3 | 17 | 15949245 | 16140836 | minus | 26 | PC | Cattle | 110 |
| TF | serotransferrin | 1 | 180137689 | 180177417 | minus | 17 | PC | Buffalo; Cattle | 129 |
| TIRAP | TIR domain containing adaptor protein | 5 | 105522721 | 105532588 | plus | 7 | PC | Cattle | 108 |
| TLR2 | toll like receptor 2 | 17 | 69069623 | 69091444 | plus | 7 | PC | Buffalo; Cattle | 28, 45 ,100 |
| TLR4 | toll like receptor 4 | 3 | 169689210 | 169700933 | plus | 5 | PC | Buffalo; Cattle | 28, 124, 130 |
| TP53 | tumor protein p53 | 3 | 36006734 | 36019283 | plus | 11 | PC | Cattle | 28, 107, 110 |
| UCP3 | uncoupling protein 3 | 16 | 31360362 | 31371780 | plus | 7 | PC | Cattle | 108 |
| VRK2 | VRK serine/threonine kinase 2 | 12 | 40396255 | 40504741 | plus | 17 | PC | Cattle | 110 |
| YES1 | YES proto-oncogene 1, Src family tyrosine kinase | 22 | 26520117 | 26584457 | plus | 14 | PC | Cattle | 108 |

**References**

107. Ron M, Israeli G, Seroussi E, Weller JI, Gregg JP, Shani M, Medrano JF. Combining mouse mammary gland gene expression and comparative mapping for the identification of candidate genes for QTL of milk production traits in cattle. BMC Genom (2007) 8:1-1. doi: https://doi.org/10.1186/1471-2164-8-183

108. Zheng J, Watson AD, Kerr DE. Genome-wide expression analysis of lipopolysaccharide-induced mastitis in a mouse model. Infect. Immun (2006) 74:1907-15. doi: 10.1128/IAI.74.3.1907-1915.2006

109. Lee JW, Bannerman DD, Paape MJ, Huang MK, Zhao X. Characterization of cytokine expression in milk somatic cells during intramammary infections with Escherichia coli or Staphylococcus aureus by real-time PCR. Vet Res (2006) 37:219-29. doi: https://doi.org/10.1051/vetres:2005051

110. Schwerin M, Czernek-Schäfer D, Goldammer T, Kata SR, Womack JE, Pareek R, Pareek C, Walawski K, Brunner RM. Application of disease-associated differentially expressed genes–Mining for functional candidate genes for mastitis resistance in cattle. Genet Sel Evol (2003) 35:1-6. doi: https://doi.org/10.1186/1297-9686-35-S1-S19

111. Pfaffl MW, Wittmann SL, Meyer HH, Bruckmaier RM. Gene expression of immunologically important factors in blood cells, milk cells, and mammary tissue of cows. J Dairy Sci (2003) 86:538-45.doi: https://doi.org/10.3168/jds.S0022-0302(03)73632-7

112. Kolbehdari D, Wang Z, Grant JR, Murdoch B, Prasad A, Xiu Z, Marques E, Stothard P, Moore SS. A whole genome scan to map QTL for milk production traits and somatic cell score in Canadian Holstein bulls. J Anim Breed Genet (2009) 126:216-27. doi: https://doi.org/10.1111/j.1439-0388.2008.00793.x

113. Pareek R, Wellnitz O, Van Dorp R, Burton J, Kerr D. Immunorelevant gene expression in LPS-challenged bovine mammary epithelial cells. J Appl Genet (2005) 46:171-7.

114. Long E, Capuco AV, Wood DL, Sonstegard T, Tomita G, Paape MJ, Zhao X. Escherichia coli induces apoptosis and proliferation of mammary cells. Cell Death Differ (2001) 8:808-16. doi: https://doi.org/10.1038/sj.cdd.4400878

115. Biendima CC, Ramos SC, Uy MR, Mingala CN. Molecular Characterization of BRCA1 as Candidate Gene Marker for Subclinical Mastitis in Dairy Water Buffaloes (Bubalus bubalis). Philipp J Sci (2017) 146:293-8.

116. Yuan Z, Chu G, Dan Y, Li J, Zhang L, Gao X, Gao H, Li J, Xu S, Liu Z. BRCA1: a new candidate gene for bovine mastitis and its association analysis between single nucleotide polymorphisms and milk somatic cell score. Mol Bio Rep (2012) 39:6625-31.doi: https://doi.org/10.1007/s11033-012-1467-5

117. Yang Y, Li Q, Ju Z, Huang J, Zhou L, Li R, Li J, Shi F, Zhong J, Wang C. Three novel single-nucleotide polymorphisms of complement component 4 gene (C4A) in Chinese Holstein cattle and their associations with milk performance traits and CH50. Vet. Immunol. Immunopathol (2012) 145:223-32. doi: https://doi.org/10.1016/j.vetimm.2011.11.010

118. Yuan ZR, Li J, Liu L, Zhang LP, Zhang LM, Chen C, Chen XJ, Gao X, Li JY, Chen JB, Gao HJ. Single nucleotide polymorphism of CACNA2D1 gene and its association with milk somatic cell score in cattle. Mol. Bio. Rep (2011) 38:5179-83. doi: https://doi.org/10.1007/s11033-010-0667-0

119. Bannerman DD, Paape MJ, Hare WR, Hope JC. Characterization of the bovine innate immune response to intramammary infection with Klebsiella pneumoniae. J Dairy Sci (2004) 87:2420-32. doi: https://doi.org/10.3168/jds.S0022-0302(04)73365-2

120. Bannerman DD, Paape MJ, Lee JW, Zhao X, Hope JC, Rainard P. Escherichia coli and Staphylococcus aureus elicit differential innate immune responses following intramammary infection. Clin. Diagn. Lab. Immunol (2004) 11:463-72. doi: 10.1128/CDLI.11.3.463-472.2004.

121. Leyva‐Baca I, Schenkel F, Sharma BS, Jansen GB, Karrow NA. Identification of single nucleotide polymorphisms in the bovine CCL2, IL8, CCR2 and IL8RA genes and their association with health and production in Canadian Holsteins Anim Genet (2007) 38:198-202. doi: https://doi.org/10.1111/j.1365-2052.2007.01588.x

122. Lee JW, Paape MJ, Elsasser TH, Zhao X. Elevated milk soluble CD14 in bovine mammary glands challenged with Escherichia coli lipopolysaccharide. J Dairy Sci (2003) 86:2382-9. doi: https://doi.org/10.3168/jds.S0022-0302(03)73832-6

123. Tabrizi AD, Batavani RA, Rezaei SA, Ahmadi M. Fibrinogen and ceruloplasmin in plasma and milk from dairy cows with subclinical and clinical mastitis. Pakistan journal of biological sciences: PJBS (2008) 11:571-6. doi: 10.3923/pjbs.2008.571.576

124. Wang XP, Xu SZ, Gao Z, Li JY, Ren HY, Luoren ZM. Cloning and SNP screening of the TLR4 gene and the association between its polymorphism and somatic cell score in dairy cattle. S. Afr. J. Anim. Sci (2008) 38:101-9.

125. Sikora KM, Magee DA, Berkowicz EW, Berry DP, Howard DJ, Mullen MP, Evans RD, MacHugh DE, Spillane C. DNA sequence polymorphisms within the bovine guanine nucleotide-binding protein Gs subunit alpha (Gsα)-encoding (GNAS) genomic imprinting domain are associated with performance traits. BMC Gen (2011) 12:1-6. doi: https://doi.org/10.1186/1471-2156-12-4

126. Wojdak-Maksymiec K, Kmiec M, Ziemak J. Associations between bovine lactoferrin gene polymorphism and somatic cell count in milk. Vet. Med (2006) 51:14.

127. Ju Z, Wang C, Wang X, Yang C, Sun Y, Jiang Q, Wang F, Li M, Zhong J, Huang J. Role of an SNP in alternative splicing of bovine NCF4 and mastitis susceptibility. PloS one. (2015) 10:e0143705. doi: https://doi.org/10.1371/journal.pone.0143705

128. Pant SD, Schenkel FS, Leyva-Baca I, Sharma BS, Karrow NA. Identification of single nucleotide polymorphisms in bovine CARD15 and their associations with health and production traits in Canadian Holsteins. BMC Gen (2007) 8:1-1. doi: https://doi.org/10.1186/1471-2164-8-421

129. Dai WT, Wang QJ, Zou YX, White RR, Liu JX, Liu HY. Comparative proteomic analysis of the lactating and nonlactating bovine mammary gland. J Dairy Sci (2017) 100:5928-35. doi: https://doi.org/10.3168/jds.2016-12366

130. Wang X, Xu S, Gao X, Ren H, Chen J. Genetic polymorphism of TLR4 gene and correlation with mastitis in cattle. J Genet Genomics (2007) 34:406-12. doi: https://doi.org/10.1016/S1673-8527(07)60044-7
